# ALDH1A3 Segregated Expression and Nucleus-Associated Proteasomal Degradation Are Common Traits of Glioblastoma Stem Cells

**DOI:** 10.3390/biomedicines10010007

**Published:** 2021-12-22

**Authors:** Julian Fauß, Bettina Sprang, Petra Leukel, Clemens Sommer, Teodora Nikolova, Florian Ringel, Ella L. Kim

**Affiliations:** 1 Laboratory of Experimental Neurooncology, Department of Neurosurgery, Johannes Gutenberg University Medical Centre, 55131 Mainz, Germany; jfauss@students.uni-mainz.de (J.F.); bettina.sprang@unimedizin-mainz.de (B.S.); 2 Institute of Neuropathology, Johannes Gutenberg University Medical Centre, 55131 Mainz, Germany; Petra.Leukel@unimedizin-mainz.de (P.L.); Clemens.Sommer@unimedizin-mainz.de (C.S.); 3 Institute of Toxicology, Johannes Gutenberg University Medical Centre, 55131 Mainz, Germany; nikolova@uni-mainz.de; 4 Department of Neurosurgery, Johannes Gutenberg University Medical Centre, 55131 Mainz, Germany; Florian.Ringel@unimedizin-mainz.de

**Keywords:** glioblastoma, glioma stem cells, ALDH1, protein turnover, proteasomal degradation

## Abstract

Aldehyde dehydrogenase 1 isoforms A1 and A3 have been implicated as functional biomarkers associated with distinct molecular subtypes of glioblastoma and glioblastoma stem cells. However, the exact roles of these isoforms in different types of glioma cells remain unclear. The purpose of this study was to dissect the association of A1 or A3 isoforms with stem and non-stem glioblastoma cells. This study has undertaken a systematic characterization of A1 and A3 proteins in glioblastoma tissues and a panel of glioblastoma stem cells using immunocytochemical and immunofluorescence staining, Western blot and the subcellular fractionation methodology. Our main findings are (i) human GSCs express uniformly ALDH1A3 but not the ALDH1A1 isoform whereas non-stem glioma cells comparably express both isoforms; (ii) there is an abundance of ALDH1A3 peptides that prevail over the full-length form in glioblastoma stem cells but not in non-stem glioma cells; (iii) full-length ALDH1A3 and ALDH1A3 peptides are spatially segregated within the cell; and (vi) the abundance of full-length ALDH1A3 and ALDH1A3 peptides is sensitive to MG132-mediated proteasomal inhibition. Our study further supports the association of ALDH1A3 with glioblastoma stem cells and provide evidence for the regulation of ALDH1A3 activities at the level of protein turnover.

## 1. Introduction

Glioblastoma (GB) is the most malignant form of brain tumors, with a final mortality rate close to 100%, less than a 10% 5-year survival rate and median survival of around 15 months [1,2]. The notorious resistance of GBs to cytotoxic and targeted therapies is related to their molecular and cellular diversity. On the molecular level, there is a multiplicity of genomic aberrations in key oncopathways with essential and redundant functions in the regulation of intrinsic and extrinsic responses in cancer cells [3]. On the cellular level, there is a remarkable degree of intratumoural diversity of cell subtypes co-existing within the same tumor. The current biological paradigm for GB is centered on so-called glioma stem cells (GSCs), implicated as the most tumorigenic type of glioma cells responsible for GB initiation and progression, before and after cytotoxic therapy [4]. Owing to their inherent plasticity, GSCs are capable of adapting to non-targeted therapies, thus defining them as the most clinically relevant target cell in GB [5]. Although some cell-membrane-associated proteins, including cell surface glycoproteins CD133/Prominin-1, CD15 and CD45, have been implicated as GSC markers, no universal marker shared by all GSCs has so far been identified [4]. There has been a growing realization in the recent years that GSCs are not uniform but comprise a heterogeneous compartment of cells that can differ with respect to the phenotypic and molecular traits [6]. Furthermore, expression levels of some GSC-associated markers are not constitutive but fluctuates during transitions between different cellular states [5,7].

There is an ongoing search for functional markers stably expressed in different molecular subtypes of GSCs. In this regard, aldehyde dehydrogenase 1 (ALDH1) has been implicated as a universal biomarker of cancer stem cells (CSCs) in different types of human cancers, including GB [8,9]. ALDH1 is a polymorphic enzyme responsible for the oxidation of aldehydes to carboxylic acids. In normal stem cells, ALDHs have a protective function in oxidative stress (by catalyzing the oxidation of endogenous and exogenous aldehydes) and regulate the biosynthesis of metabolites crucial for the central nervous system development and homeostasis [10]. Several lines of evidence indicate that ALDH1 enzymes have important roles in promoting GB growth: (i) high expression of ALDH1 correlates with a higher grade of malignancy and poorer prognosis [11,12,13]; (ii) pharmacologic or shRNA-mediated inhibition of ALDH1 sensitizes conventional glioma cell lines to TMZ [12,13]; and (iii) ALDH1 expression is positively associated with the tumor-propagating potential in GSCs [9,14,15,16]. Isoforms ALDH1A1 and ALDH1A3 have particularly been implicated in GB although the exact roles of these isoforms in different types of glioma cells remain unclear. The ALDH1A1 isoform is a 501-amino acid NAD-dependent aldehyde dehydrogenase encoded by a gene comprised of 13 exons spanning ~53 kb on chromosomes 9(9q21.13) (http://atlasgeneticsoncology.org/Genes/GC_ALDH1A1.html accessed on 17 December 2021). ALDH1A3-coding gene is located on chromosome 15(15q26.3), comprises 13 exons spanning ~36 kb and encodes for a 512-amino-acid NAD-dependent aldehyde dehydrogenase (http://atlasgeneticsoncology.org/Genes/GC_ALDH1A3.html accessed on 17 December 2021).

ALDH1A1 and ALDH1A3 isoforms are topologically similar [17] but seem to be functionally non-redundant and may have distinct roles in brain malignancies. Recent studies indicate that ALDH1A1 or ALDH1A3 expression may be associated with distinct molecular subtypes of GB, with ALDH1A3 being the mesenchymal subtype [15,16,18] and ALDH1A1 associated with classical GBs [13], showing a better response to combined radiochemotherapy compared to mesenchymal GBs [19].

An association between ALDH1 expression and a particular molecular subtype of GB has been derived from the results of genome-wide profiling of transcriptomic or (epi)genomic landscapes using whole-tissue tumor specimens [19,20]. While revealing an association between ALDH1A1, ALDH1A3 and a particular GB subtype, whole-tissue analyses do not allow to discern unequivocally the association of different ALDH1 isoforms with GSCs, which constitute a distinct and the most clinically relevant type of GB cell. Furthermore, a comparative analysis of the abundance and patterns of ALDH1 proteins across different types of GB cells, such as non-stem glioma cells and GSCs, has not been performed. Recent developments indicate that protein turnover plays important roles in the maintenance of steady-state levels and activities of ALDH1 proteins. It has been shown that increased levels of ALDH1A3 in mesenchymal GSCs is primarily achieved via the enhanced expression of ubiquitin-specific proteinase USP9x which has been identified as a specific regulator of ALDH1A3 [21]. These intriguing findings indicate that post-transcriptional modifications play important roles in the regulation of ALDH1 activities and urge a further investigation of ALDH1 isoforms at the protein level. As many previous investigations related to ALDH1 proteins have been performed in glioma cell lines that lack key properties of stem cells, it is also important to clarify whether and to what extent the traits established in non-stem glioma cells hold for GSCs. In the current study, ALDH1 isoforms were characterized at the protein level in a panel of GSCs derived from newly diagnosed or recurrent GBs.

## 2. Materials and Methods

### 2.1. GB Tissues and GSCs

Tumor samples were collected from patients diagnosed with newly diagnosed or recurrent GB and undergoing treatment at the Johannes-Gutenberg University Medical Center Mainz (UMM). Freshly resected tumor tissue was used for GSCs isolation in accordance with the approval of ethics committee (No. 837.178.17(11012) and patient’s informed consent. Human GSCs were isolated as previously described [22,23,24]. GSCs were maintained in NeuroBasal complete medium (Neurobasal^TM-^A + B27 supplement, Gibco, Life Technologies, Darmstadt, Germany) containing the self-renewal promoting factors bFGF (basic fibroblast growth factor, 10 ng/mL) and EGF (epidermanl growth factor, 20 ng/mL) (Biochrom GmbH, Berlin, Germany). Serum-dependent glioma cell lines U87 and LN229 were purchased from the American Type Cell Culture Collection (ATCC) and propagated in DMEM medium supplemented with 5% fetal calf serum.

### 2.2. Cell Based Assays

For self-renewal evaluation, the extreme limited dilution assay (ELDA, http://bioinf.wehi.edu.au/software/elda/ accessed on 17 December 2021) was performed using the following conditions: GSCs were plated at clonal densities (0.625 to 10 cells/mL) in NeuroBasal complete medium and incubated for 28–42 days to enable the formation of clonal gliomaspheres counted using the phase contrast microscope. Stem cell frequency was determined with the ELDA webtool [25]. Evaluation of the differentiation potential was performed by comparative immunophenotyping of GSCs cultured under either a self-renewal-promoting (bFGF+/EGF+) or differentiation-inducing (bFGF and EGF withdrawal) culture condition. GSCs were plated at 30,000–50,000 on glass coverslips coated with poly-L-ornithine hydrobromide (15 µg/mL, Sigma Aldrich, Munich, Germany) and incubated for 7 to 10 days prior to cells fixation. Fixed cells were analyzed by immunofluorescence staining for lineage specific markers.

### 2.3. Immunohistochemistry

Formalin-fixed and paraffin-embedded tissue sections of 4 µm were dewaxed and subjected to antigen retrieval with the EnVision FLEX Target Retrieval reagent, high pH (DAKO, Hamburg, Germany). After the blocking step using peroxidase blocking solution (DAKO, Germany), immunohistochemical staining with anti-ALDH1 (clone 44, 611,194 BD Biosciences, dilution 1:000) or anti-ALDH1A3 (ab129815, Abcam, Cambridge UK, dilution 1:300) antibodies was done in an automated stainer (Dako Autostainer Plus, DAKO). Visualization of immunoreactivity was performed using the universal immuno-enzyme polymer method (Nichirei Biosciences Inc., Tokyo, Japan). Sections were developed in diaminobenzidine (Lab Vision Corporation, Fremont, CA, USA). As a control, a subset of slides was processed in parallel under the identical conditions except for the omission of anti-ALDH1 antibodies. Immunostaining results were evaluated by an experienced neuropathologist (CJS).

### 2.4. Immunofluorescence Staining

Cell were incubated in 4% paraformaldehyde/PBS (Merck KGaA, Darmstadt, Germany) for five minutes at room temperature followed by fixation with the methanol + acetone mix (50% *v*/*v*) at −20° C. Prior to immunofluorescence staining, fixed cells were re-hydrated and permeabilized by incubation in 0.2% Triton X-100/PBS (Sigma-Aldrich Chemie GmbH, Taufkirchen, Germany) for five minutes at room temperature. Anti-ALDH1 antibodies used were ab52492 (anti-ALDH1A1, Abcam,), MA5-25528 (anti-ALDH1A3, Thermo Fischer Scientific, Darmstadt, Germany), PA5-29188 (anti-ALDH1A3, InVitrogen, ThermoFischer Scientific, Darmstadt Germany), ab129815 (anti-ALDH1A3, Abcam, Cambridge, UK). Anti-nestin and anti-GFAP antibodies were from Abcam (ab22035) or DAKO (Z0334), respectively. Fluorescently labeled secondary antibodies (anti-mouse Alexa Fluor 488 or anti-rabbit Alexa Fluor 555) were purchased from Thermo Fisher Scientific. Nuclear counterstaining was performed by using DAPI (4,6-diamidino-2-phenylindole, Sigma) for immunofluorescence microscopy or To-Pro-3 (Invitrogen, Thermo Fischer Scientific) for laser scanning microscopy. Antibodies specificity was confirmed by staining of cells with secondary antibodies alone. Image acquisition was performed by using a Leica DM IRB immunofluorescence microscope equipped with LAS X software from Leica Microsystems, Wetzlar, Germany or laser scanning microscopy (LSM710, Carl Zeiss MicroImaging) equipped with ZEN 2009 software (Zeiss Microscopy, Oberkochen Germany).

### 2.5. Western Blot and Subcellular Fractionation

Whole-cell protein extracts were prepared by disrupting the cell pellet in Lysis Buffer (20 mM Tris, 1 mM EDTA, 1 mM β-Mercaptoethanol, 5% Glycerine, pH 8.5) supplemented with an EDTA-free protease inhibitor cocktail (Roche^®^Life Science Products) using sonification. Proteins (20–80 µg per well) were separated by sodium dodecyl sulphate polyacrylamide gel electrophoresis (SDS-PAGE) and transferred overnight at 100 mA in blotting buffer (0.025 M Tris, 0.192 M Glycine, 20% Methanol) onto a nitrocellulose membrane. For subcellular fractionation, 5 × 10^7^ cells were seeded three days before fractionation. After collection by centrifugation cells were washed twice with ice-cold PBS and resuspended in cell membrane permeabilization buffer (10 mM HEPES, pH 7.9; 10 mM KCl, 1.5 mM MgCl2, 0.34 M sucrose, glycerol 10%) supplemented with protease inhibitors. After a 10 min incubation on ice, the cell lysate was centrifuged at 1300× *g* for 5 min at +4 °C. The supernatant (whole lysate) was transferred to a new tube and subjected to clarification by centrifugation at 17,000× *g* for 15 min at +4 °C. The clarified supernatant (cytosolic fraction) was transferred into a new tube and used for Western blot analyses. Nuclear pellets were washed extensively in cell membrane permeabilization buffer, resuspended in nuclear lysis buffer (3 mM EDTA, pH 8.0; 0.2 mM EGTA; 1 mM DTT) supplemented with protease inhibitors and centrifuged at 1300× *g* for 5 min at +4 °C. After centrifugation the supernatant was discarded, and the nuclear-containing pellet disrupted by eight rounds of ultrasound sonication in a 30 s on/30 s off regimen. After sonification, nuclear fractions were clarified by centrifugation and analyzed by Western blot using pre-cast tris-glycine gels (4–12% or 12%, Bio-Rad, Feldkirchen, Germany). For proteasomal inhibition, 2 × 10^6^ cells were treated with either 25 µM MG132 (Merck KGaA, Darmstadt, Germany) or mock-treated (DMSO) for 24 h. After the treatment, cells were collected by centrifugation, washed twice with ice-cold PBS and subjected to analyses by Western blot. Protein expression was analyzed via densitometry using ImageJ. The expression of ALDH1A3-FL or ALDH1A3 peptides was normalized to actin. The mock-treated control was set to 100 percent.

## 3. Results

### 3.1. Expression Patterns of ALDH1A3 and ALDH1A1 in GBs and Patient-Derived GSCs

**Figure 1:**
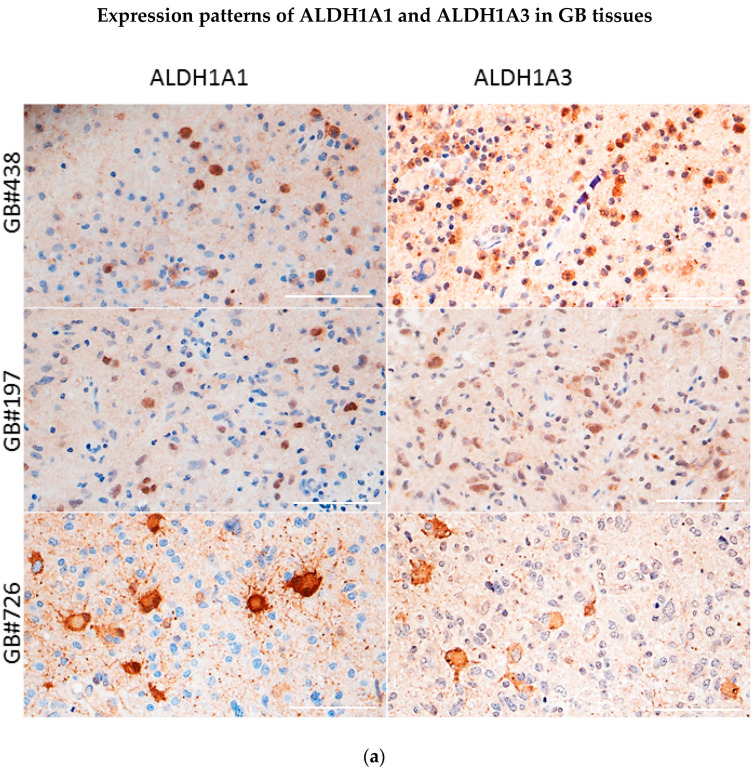

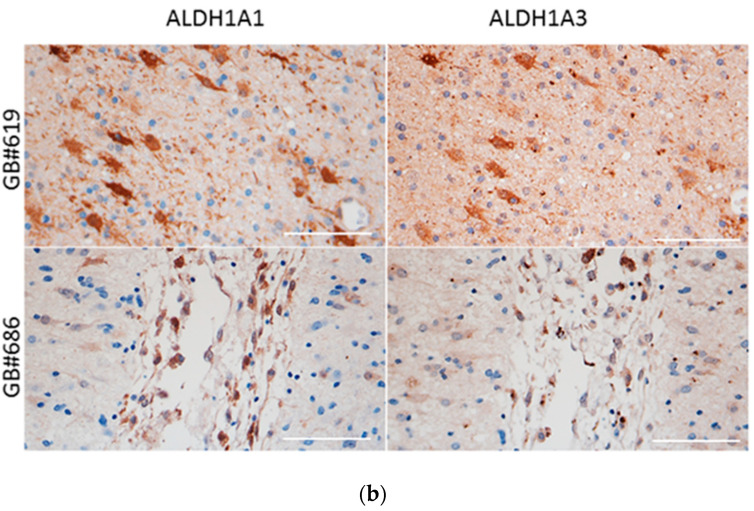
Immunohistochemical co-staining of GB tissues reveals either segregated (**a**) or concomitant (**b**) expression of ALDH1A1 and ALDH1A3 in tumor cells. Magnification 40×. Scale bar, 100 µm.

Previous investigations of ALDH1 isoforms in GB have relied on expression patterns identified by using non-selective antibodies that recognize both ALDH1A1 and ALDH1A3 isoforms. To clarify if the two isoforms are co- or differentially expressed in GBs, we used antibodies specific for either the ALDH1A1 or ALDH1A3 isoform. IHC assessments of ten GBs reveal heterogeneous patterns characterized by either segregated or concomitant expression of ALDH1A1 and ALDH1A3 in GB cells (Figure 1, data shown for five representative tumors). In some cases, both patterns could be found within the same tumor (data not shown). To determine which of the two patterns (simultaneous or segregated) is associated with GSCs, we analyzed the ALDH1A1 or ALDH1A3 isoforms in a panel of ten cultures of GSCs isolated from newly diagnosed or recurrent GBs and maintained exclusively in the absence of serum, an experimental condition that favors propagation of undifferentiated GSCs in vitro [26]. All GSC cultures used in this study were tested for the self-renewal propensity and degree of inherent phenotypic plasticity, as exemplified in Figure S1. In parallel with GSCs, serum-grown glioma cell lines LN229 and U87 were used as experimental models of glioma cells lacking stemness properties. Concordant with their lack of stemness, LN229 and U87 cells are incapable of self-renewal and unable to undergo morphophenotypic changes upon exposure to differentiation-inducing conditions (data not shown). At the molecular level, the difference between GSCs and non-stem glioma cells manifests in the expression of stem cell marker.

**Figure 2:**
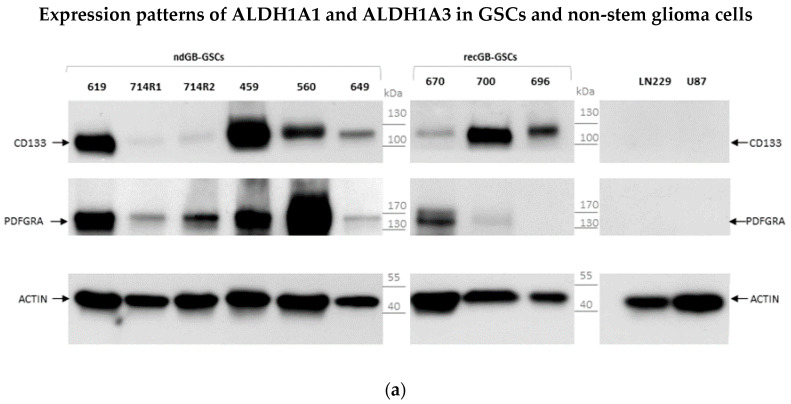

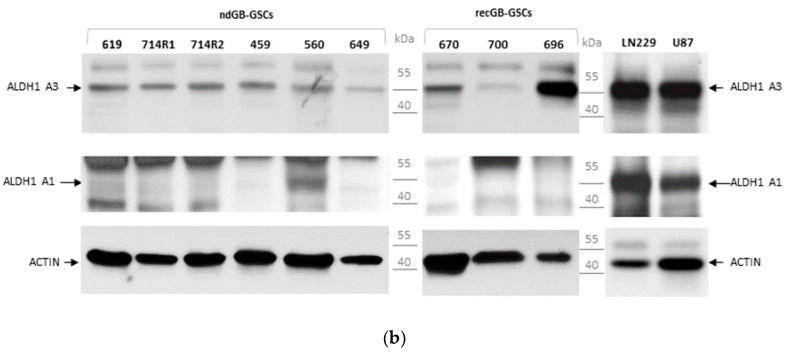
Western blot analyses of total protein content in patient-derived GSCs and non-stem glioma cells U87 and LN229. (**a**) Comparative assessments for GSC markers CD133 and PDGFRα. Patient-derived GSCs express CD133 and PDGFRα show the expression of either CD133 or PDGFRα, or both, whereas non-stem glioma cells U87 and LN229 are devoid of either CD133 or PDGFRα. (**b**) Comparative assessments of ALDH1A1 and ALDH1A3 in patient-derived GSCs and non-stem glioma cells U87 and LN229. Non-stem glioma cells express both ALDH1A1 and ALDH1A3 isoforms, whereas patient-derived GSCs show preferential expression of ALDH1A3.

CD133 was positively associated with GB aggressiveness [25,27] and PDGFRα, one of the critical genes involved in glioma progression and the second most frequently overexpressed TRK in GB [18]. While GSCs express at least one of these markers, serum-grown cell lines express neither CD133 nor PDGFRα (Figure 2a). We next characterized the expression of ALDH1 isoforms in GSCs along with serum-grown glioma cell lines LN229 and U87 in which ALHD1 expression has been investigated extensively. In accordance with previous studies, both ALDH1A1 and ALDH1A3 isoforms were abundantly expressed in LN229 and U87 cells (Figure 2b).

**Figure 3:**
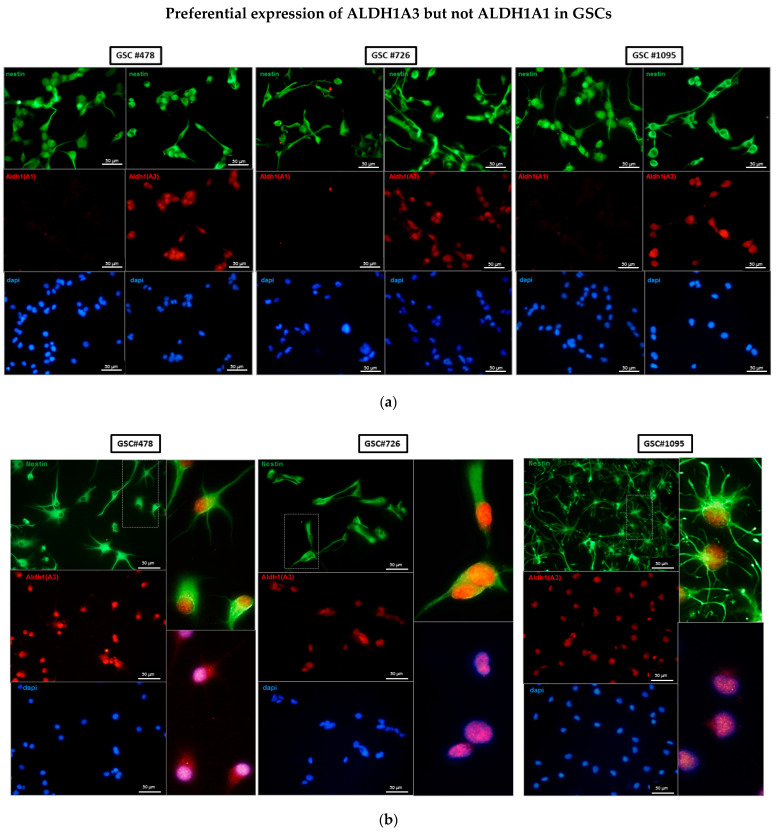
Assessments of ALDH1A1 and ALDH1A3 expression in GSCs by immunofluorescence staining. (**a**) GSCs cultured under self-renewal-promoting conditions and co-stained with anti-ALDH1A1 or anti-ALDH1A3 (red) and anti-nestin (green) antibodies. Counterstaining with DAPI. ALDH1A3 but not ALDH1A1 expression is readily detectable in self-renewing GSCs. Magnification 20×. Scale bars corresponds to 50 µM. (**b**) ALDH1A3 expression persists in GSCs under differentiation-inducing condition. GSCs were propagated under differentiation-inducing condition (withdrawal of self-renewal-promoting factors bFGF and EGF) and co-stained with anti-ALDH1A3 (red) and anti-nestin (green) antibodies. Counterstaining by DAPI. Magnification 20×. Scale bars correspond to 50 µM. Insets show enlarged images of individual cells demarcated by broken lines. The ALDH1A3 signal is readily seen in both the nuclear and cytoplasmic compartments.

In GSCs, a more segregated pattern of ALDH1A3 and ALDH1A1 was observed. In contrast to the ALDH1A3 isoform clearly detectable in all GSC lines tested (top panel), the expression of ALDH1A1 was highly variable (middle panel). Out of nine GSC lines tested only one (GSC#560) showed appreciable levels of ALDH1A1. These data suggest that the concomitant expression of both ALDH1A1 and ALDH1A3 is primarily associated with non-stem glioma cells, whereas the expression of ALDH1A3 but not ALDH1A1 appears to be a phenotypic trait conserved across GSCs. Concordant with this conclusion immunofluorescence assessments also showed a nearly homogeneous staining pattern for ALDH1A3 accompanied by the virtually complete lack of ALDH1A1 in all GSCs analyzed in this study (typical results are shown in Figure 3). Notably, ALDH1A3 expression was comparable between GSCs that have been propagated either in the presence or absence of the self-renewal-promoting factors bFGF and EGF (Figure 3a). As bFGF and EGF withdrawal is known to induce in vitro differentiation of differentiation-capable GSCs, these data suggest that ALDH1A3 expression cannot be attributed to a particular cellular state of GSCs.

### 3.2. Lack of Association between ALDH1A3 and Molecular Markers of the Proneural or Proliferative Molecular Subtypes

**Figure 4:**
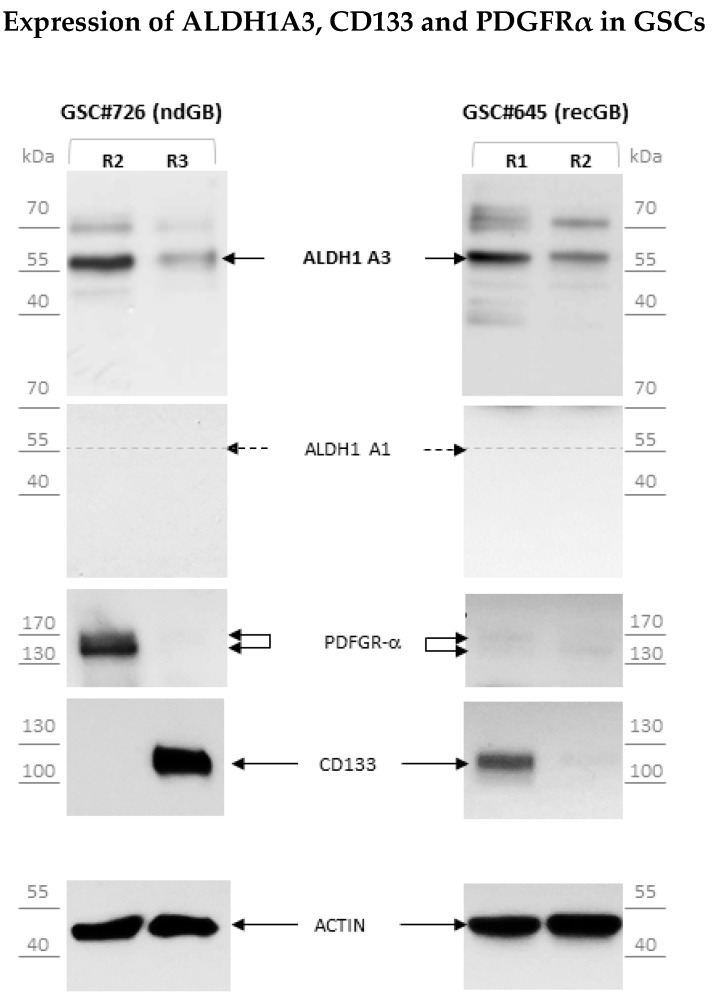
Comparative assessments of the CD133, PDGFRa and ALDH1 isoforms in isogenic GSCs by Western blot. “R1”, “R2” and “R3” designate different tumor regions from which isogenic GSCs were isolated. Isogenic GSCs show preferential expression of ALDH1A3 but not ALDH1A1. Considerable intratumoral variations in the levels of CD133 or PDFGRα do not mirror variations in the levels of ALDH1A3.

Increased expression of ALDH1A3 has been associated with mesenchymal GBs. We next asked whether there is a correlation between ALDH1A3 expression and CD133 or PDGFRα, the markers of proliferative and proneural GBs, respectively [19]. We addressed this question in isogenic GSCs that have been isolated from different regions of the same tumor [24] and therefore have a common genetic background. The association between CD133 and PDGFRα with distinct molecular subtypes of GB and supports previous findings that different molecular subtypes can co-exist within the same tumor [24,28,29]. Isogenic GSCs derived from either newly diagnosed or recurrent GB showed mutually exclusive expression of these markers (Figure 4). Interestingly, either CD133- or PDGFRα-expressing GSCs showed comparable levels of ALDH1A3, suggesting that the latter may be expressed across molecularly distinct subtypes of GSCs.

### 3.3. Abundant Expression of Truncated ALDH1A3 Peptides in GSCs

**Figure 5:**
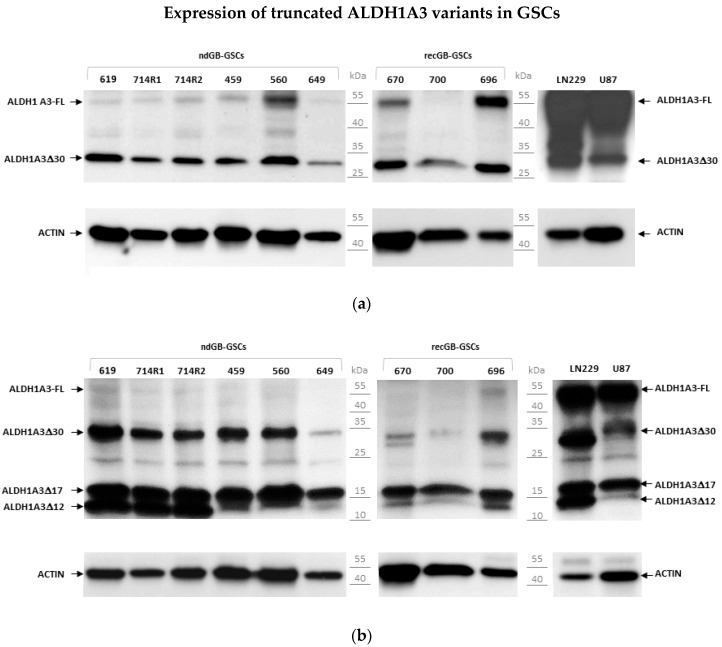
Western blot assessments of ALDH1A3 by internally binding anti-ALDH1A3 antibodies: (**a**) pattern revealed by antibody PA5-29188 (aa126-423); (**b**) pattern revealed by antibody ab129815 (aa100-200). The abundance of truncated ALDH1A3 proteins relative to the full-length ALDH1A3 (ALDH1A3-FL) is much greater in GSCs than in non-stem glioma cells U87 and LN229.

Several truncated variants of human ALDH1A3 lacking either N- or C-terminal portions of the protein have been reported (https://www.uniprot.org/uniprot, accessed on 17 December 2021, entry numbers P47895-1, H0Y2 × 5, H0YNQ3, H0YKF9, H0YLT1). In our initial assessments of ALDH1A3 by Western blot, we used a dual epitope-binding antibody MA5-25528. We considered the possibility that shorter variants of ALDH1A3 may have escaped detection with the antibody MA5-25528, which requires the presence of two binding sites located at the N- and C-termini of the ALDH1A3 protein (Figure S2). To test this possibility, ALDH1A3 expression was further assessed by using anti-ALDH1A3 antibodies that bind to epitopes located within internal regions of the ALDH1A3 protein. Assessments using antibody PA5-29188, which binds to the ALDH1A3 central region (Figure S2), revealed abundant amounts of a peptide with a lower molecular weight (~30 kDa) than the full-length ALDH1A3 protein (ALDH1A3-FL), which has 56 kDa (Figure 5a).

As PA5-29188 antibody binds in the middle of ALDH1A3 (Figure S2), these results suggest that the 30 kDa peptide (termed provisionally as “ALDH1A3∆30 “) is lacking either the N- or C-terminal portion of ALDH1A3. The ALDH1A3∆30 peptide was also recognized by another anti-ALDH1A3 antibody, ab129815, which binds to the N-terminal region spanning 100–200 amino acid residues of the ALDH1A3 protein (Figure S2 and Figure 5b). Furthermore, in addition to ALDH1A3∆30, ab129815 revealed two additional peptides migrating with the apparent molecular weight of 17 and 12 kDa (termed as ALDH1A3∆17, and ALDH1A3∆12, respectively, Figure 5b). Notably, although ALDH1A3∆30, ALDH1A3∆17 and ALDH1A3∆12 could also be detected in non-stem glioma lines LN229 and U87 (Figure 5), their proportions relatively to ALDH1A3-FL differed between GSCs and non-stem glioma cells. ALDH1A3-FL was found to prevail over truncated variants in non-stem glioma cells but not in GSCs, showing a clear predominance of truncated variants over the full-length protein (Figure 5).

### 3.4. Segregated Subcellular Localization of ALDH1A3-FL and Truncated ALDH1A3 Peptides

**Figure 6:**
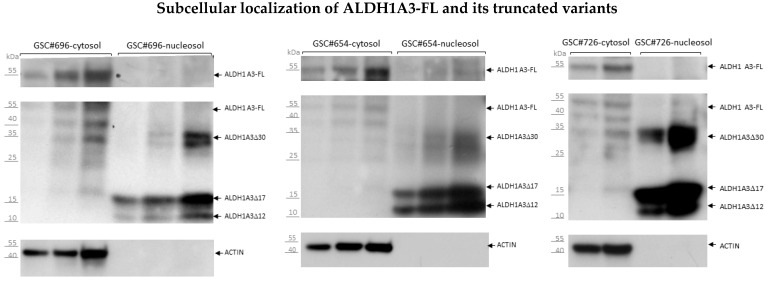
Western blot analysis of the cytosolic and nuclear fractions. Increasing protein amounts (20–80 µg per well) of each fraction were loaded and probed sequentially with ab129815 (for ALDH1A3 peptides) followed by probing with antibody MA5-25528 (for ALDH1A3-FL). After probing with ALDH1A3 antibodies, the membranes were stripped and probed for actin to assure lack of contamination with cytosolic proteins in the nuclear fractions.

In the course of our immunostaining analyses we noticed that internal anti-ALDH1A3 antibodies generate an intense signal in the nuclear compartment in both cultured GSCs (Figure 3b) and whole GB tissues (Figure S3). To determine if the nuclear localization is associated with ALDH1A3-FL or its truncated variants we performed subcellular fractionation experiments. The purity of the nuclear fractions was confirmed by the absence of cytoplasmic (actin-β) or plasma membrane (CD133) resident proteins and enrichment for histone H2B, as exemplified in Figure S4. Cytosolic and nuclear fractions was further assessed for ALDH1A3-FL and ALDH1A3 peptides. All GSCs analyzed in this study (ten GSC lines from either newly diagnosed or recurrent GBs) showed a segregated pattern of subcellular distribution for ALDH1A3-FL and ALDH1A3 peptides (Figure 6, representative data from three GSC lines shown). As expected, ALDH1A3-FL was virtually absent in the nuclear compartment and localized exclusively in the cytoplasm (compare panels “cytosol” and “nucleosol”), whereas the ALDH1A3∆30, ALDH1A3∆17 and ALDH1A3∆12 variants were found predominantly in the nuclear fraction.

### 3.5. Proteolytic Cleavage as the Mechanism of Origin for ALDH1A3 Peptides

**Figure 7:**
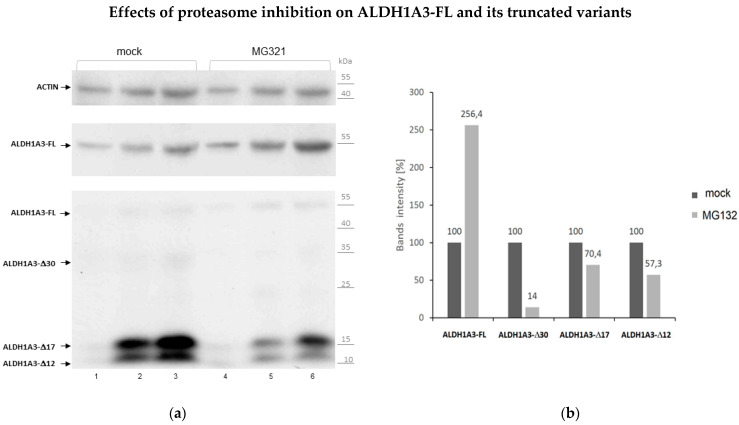
Effects of proteasome inhibition by MG132 on the steady-state levels of ALDH1A3-FL and its truncated variants. (**a**) Western blot assessments of whole lysates of GSC#726/R1 cells treated with MG132 or DMSO (mock control). Increasing protein amounts (40, 60 and 80 µg per well) were loaded in lanes 1&4, 2&5 and 3&6, respectively. After probing with ALDH1A3 antibodies, the membrane was stripped and probed for actin. (**b**) Graphical presentation of the normalized data. The intensity of the bands corresponding to ALDH1A3-FL, ALDH1A3∆17 or ALDH1A3∆12 was quantified by densitometry and normalized to actin. The mock-treated control was set to 100 (black bars). Grey bars show the mean change in the abundance of ALDH1A3-FL, ALDH1A3∆17 or ALDH1A3∆12 after MG132 treatment compared to mock-treated control samples. Proteasome inhibition leads to a decrease in the abundance of truncated ALDH1A3 proteins and increase of the full-length ALDH1A3.

Considering that ALDH1 proteins lack the nuclear localization signal [30] and that ALDH1A3 turnover is regulated by the ubiquitin-proteasome system [21], we considered the possibility that ALDH1A3 peptides are the products of proteasomal degradation. To address this hypothesis, we investigated the effect of the ubiquitin-proteasome inhibitor MG132 on the levels of ALDH1A3-FL and ALDH1A3 peptides in GSCs. The efficacy of MG132 treatment was confirmed by monitoring the levels of tumor suppressor p53, a bona fide target for proteasomal degradation (Figure S5). Analyses of ALDH1A3 proteins showed that MG132 treatment leads to a considerable increase in the ALDH1A3-FL levels whereas the levels of the ALDH1A3∆30, ALDH1A3∆17 or ALDH1A3∆12 variants were reduced after the treatment with MG132 (Figure 7). Parallel but nonreciprocal changes in the abundance of ALDH1A3-FL or its shorter variants upon proteasomal inhibition is consistent with the interpretation that the latter ones originate from proteolytic cleavage of ALDH1A3-FL.

## 4. Discussion

This study addresses the association between the ALDH1 isoforms A1 and A3, implicated as the markers of GB aggressiveness and patient-derived GSCs. We provide evidence that human GSCs express uniformly ALDH1A3 but not the ALDH1A1 isoform, whereas non-stem glioma cells express both isoforms at comparable levels (Figure 2b and Figure 3). Although the expression of ALDH1A3 is not restricted solely to GSCs, the composite ALDH1A3+/ALDH1A1-phenotype is a characteristic trait of GSCs whereas the concomitant expression of both ALDH1A3 and ALDH1A1 isoforms appears to be associated with glioma cells lacking stemness. Compared to other GSC markers, such as CD133, ALDH1A3 expression is less variable and persists in different cellular states of GSCs. Our investigations reveal for the first time the abundance of short ALDH1A3 peptides that appear prevailing over the full-length form in GSCs but not in non-stem glioma cells (Figure 5). Furthermore, we found that the full-length ALDH1A3 and ALDH1A3 peptides are spatially segregated within the cell with ALDH1A3-FL residing in the cytosol while ALDH1A3-derived peptides are almost exclusively localized in the nucleoplasm (Figure 6). We provide evidence that the relative proportion of ALDH1A3-FL and ALDH1A3 peptides is sensitive to the proteasomal inhibitor MG132 (Figure 7). Considering that ALDH1A3 turnover is regulated via the ubiquitin-proteasome system [21] and that the nucleus is an important site for the proteasome-mediated degradation of cytoplasmic proteins [29,31], our findings indicate that ALDH1A3 undergoes constant turnover in GSCs, with the ultimate breakdown of the ALDH1A3 protein taking place in the nucleus (Figure 6 and Figure 7). Chen et al. have previously demonstrated that overexpression of ubiquitin-specific proteinase 9X leads to stabilization of recombinant ALDH1A3 protein in mesenchymal GSCs [21]. These findings have led to the formulation of a novel hypothesis that proteolytic degradation is an important mechanism regulating the ALDH1A3 steady-state levels and activities in GSCs. However, the evidence to support the proteolytic cleavage of endogenous ALDH1A3 was still missing. By demonstrating the abundance of ALDH1A3 peptides naturally occurring in human GSCs, our study not only provides such evidence but also provides further insights into the mechanism of ALDH1A3 turnover via proteasome-dependent breakdown in the nucleus. Although the role of the nucleus-based quality control of cytoplasmic proteins in mammalian cells has not yet been fully elucidated, the principal possibility of cytoplasmic proteins degradation in the nucleus has been shown in human cells [32,33]. Although we favor the interpretation that proteasomal degradation in the nucleus is the mechanism for generating truncated ALDH1A3 peptides, it also cannot be ruled out that ALDH1A3 fragments may be hauled to the nucleus by some nuclear proteins or translocated by passive diffusion [34]. Further in-depth investigations are needed to unequivocally clarify the role of nuclear proteasomes in the maintenance of ALDH1A3.

Another uncertainty is whether the truncated ALDH1A3 proteins derive from the wild-type ALDH1A3 protein. In this regard, it is noteworthy that mutant ALDH1A3 proteins can be generated via the aberrant alternative splicing or gene fusions involving the ALDH1A3 gene. Indeed, a number of intronic mutations, leading to the ALDH1A3 aberrant splicing [35,36] as well as ALDH1A3 fusion transcripts (http://atlasgeneticsoncology.org/Genes/GC_ALDH1A3.html accessed on 17 December 2021), have been identified. Interestingly, one of the known fusion partners of ALDH1A3 is USP25, a ubiquitin-dependent protease, which localizes in both nucleus and cytoplasm and has been implicated as a tumor-promoting factor in different types of human cancers [37]. In order to unequivocally identify the precursor protein for truncated ALDH1A3 peptides the determination of their amino acid composition should be the next step.

The abundance of ALDH1A3 peptides in GSCs raises several implications for the further refinement of diagnostic predictions based on assessments of ALDH1A3 in GB specimens: (i) scoring for the composite ALDH1A3+/ALDH1A1- phenotype might predict the degree of aggressiveness more precisely than solo ALDH1A3+ scores, which do not enable to discriminate between GSCs and non-stem glioma cells; (ii) the results of histologic evaluations may be obscured when using antibodies that do not discriminate between the full-length ALDH1A3 and its truncated variants; and (iii) abundant contribution of ALDH1A3 structural variants should be considered when scoring ALDH1A3 expression in GB tissues/GSCs.

Collectively, our findings urge to further delineate the protein modifiers involved in ALDH1A3 turnover, which may provide new insights for further testing the hypothesis that targeting ALDH1A3 may be effective in reducing the tumor-promoting potential of GSCs [38,39].

## Data Availability

Not applicable.

## Supplementary Materials

The following are available online at https://www.mdpi.com/article/10.3390/biomedicines10010007/s1, Figure S1: Evaluation of stemness-associated properties in patient-derived GSCs.; Figure S2: Graphical presentation of binding sites for anti-ALDH1A3 antibodies used in the study.; Figure S3: Immunofluorescence staining of GB tissues.; Figure S4: Efficacy of subcellular fractionation.; Figure S5: Efficacy of MG132-mediated proteasome inhibition.
