# Supplementary material for: ALDH1A3 Segregated Expression and Nucleus-Associated Proteasomal Degradation Are Common Traits of Glioblastoma Stem Cells
Source: Biomedicines. 2021 Dec 22;10(1):7. doi: 10.3390/biomedicines10010007 (PMC8772809; doi:10.3390/biomedicines10010007)
Supplement: Supplementary file 1 [file biomedicines-10-00007-s001.zip › biomedicines-1514373-sup.pdf]

Figure S1

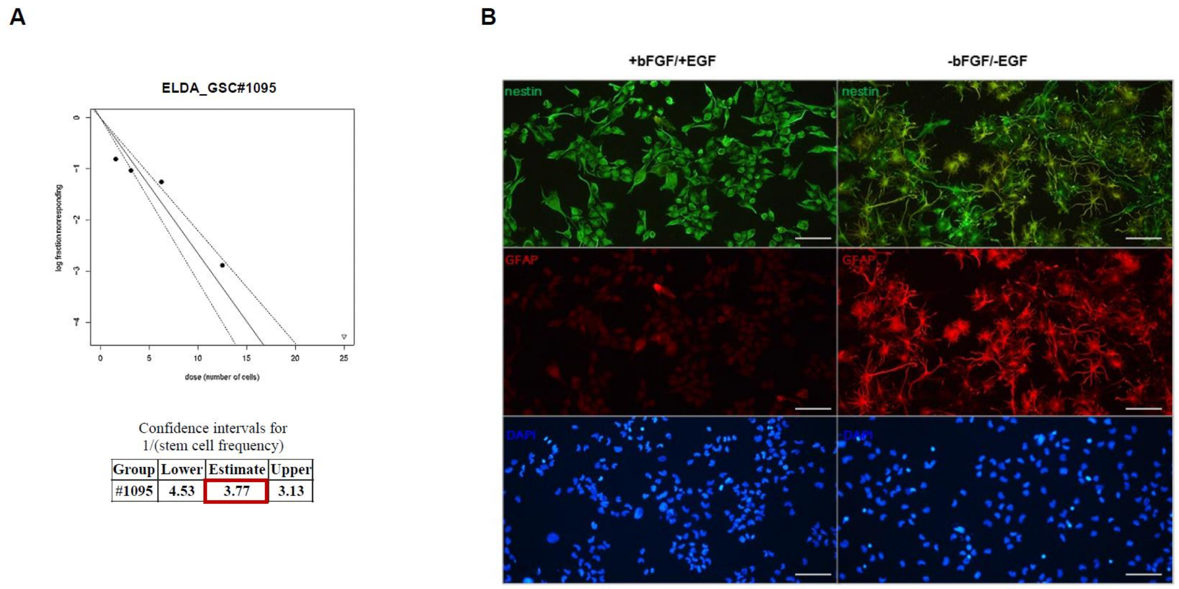

Confidence intervals for
1/(stem cell frequency)

Group	Lower	Estimate	Upper
#1095	4.53	3.77	3.13

Figure S1. Evaluation of stemness-associated properties. *A*, estimation of self-renewal by ELDA. *A*, graphical presentation of ELDA results. Y axis ("log fraction nonresponding") shows the frequency of cells uncapable of forming clonal spheres determined at varying cell densities. X axis ("dose") corresponds to cells number per mL. The data values with zero negative responses are represented by a down-pointing triangle. The dotted lines give 95% confidence interval. Red-framed values correspond to estimated stem cell frequency calculated by using the ELDA algorithm. *B*, evaluation of the differentiation potential by comparative immunophenotyping. Cells were cultured either in the presence or absence of self-renewal promoting factors bFGF and EGF and analyzed for morphological changes and expression of the astrocytic differentiation marker GFAP (red) or neural stem cell/progenitor marker nestin (green) by immunofluorescence staining. Counterstaining by DAPI. Magnification 20×. Bar scales 100 µm.

Figure S2

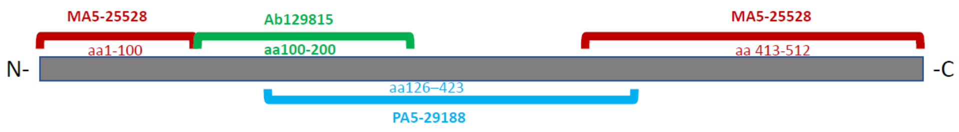

Figure S2. Graphical presentation of binding sites for anti-ALDH1A3 antibodies used in the study. Numbers indicate amino acid residues corresponding to antibodies binding epitopes.

A **Figure S3**

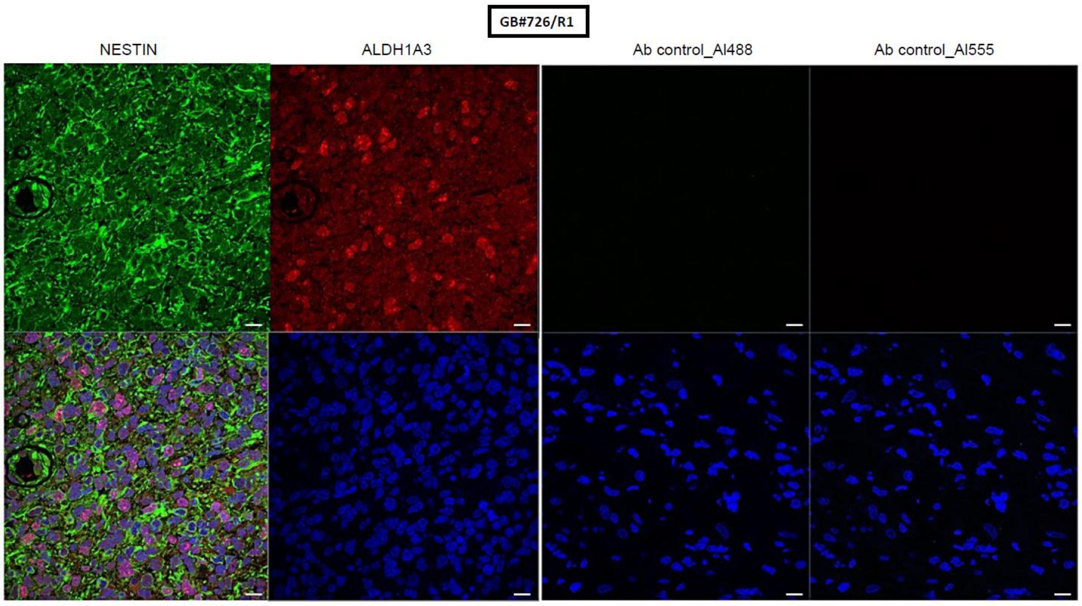

B **Figure S3**

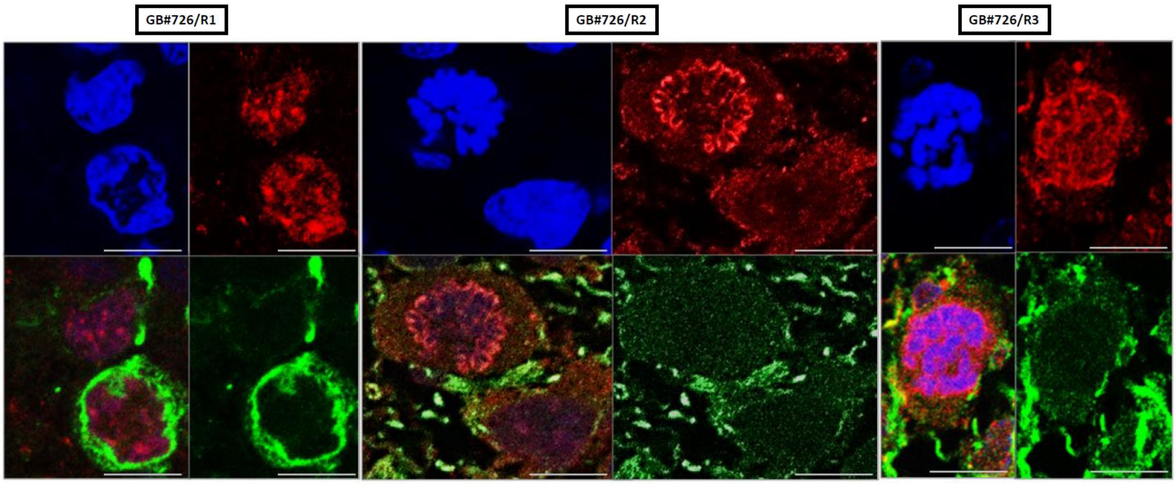

Figure S3. Immunofluorescence staining of GB tissues. Imaging by laser scanning microscopy. *A*, micrographs of GB#726 co-stained with anti-nestin (green) and anti-ALDH1A3 antibody ab129815 (red). Nuclear staining by To-Pro-3 (Invitrogen Thermo Fischer Scientific). Controls for antibodies specificity are shown in the right panel. Magnification 20x. Bar scales 20 µm *B*, Single tumor cells showing both the nuclear and cytoplasmic staining with anti-ALDH1A3 antibody ab129815. Magnification 63x. Bar scales 10 µm. “R1”, “R2” and “R3” correspond to different regions from GB#726.

Figure S4

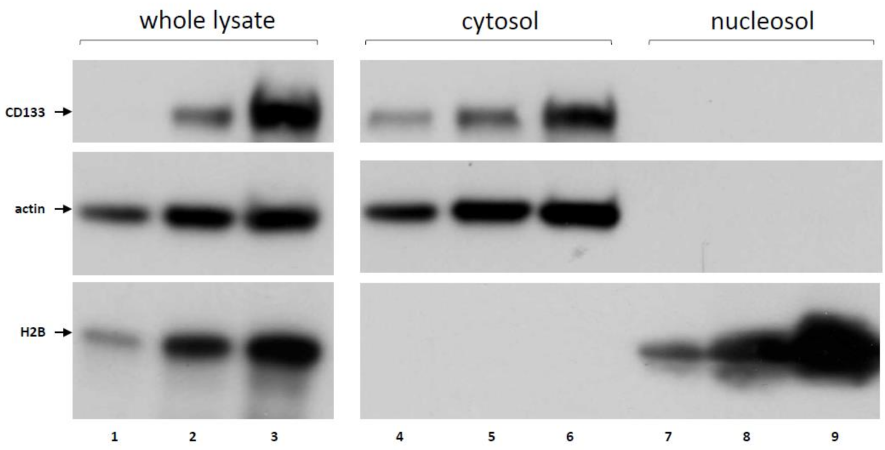

Figure S4. Efficacy of subcellular fractionation using GSC line #726/R1. Western blot analysis to ascertain the degree of enrichment for cytosolic and nuclear proteins. Increasing protein amounts (20-80 µg per well) of the whole lysate (lanes 1-3), cytosolic (lanes 4-6) or nuclear (lanes 7-9) fractions were loaded and analyzed for the content of non-nuclear (CD133, actin) and nuclear (histone H2B) resident proteins.

Figure S5

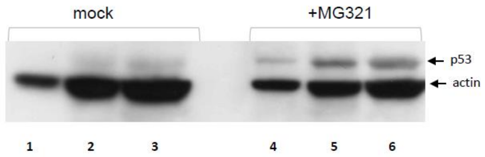

Figure S5. Efficacy of MG132-mediated proteasome inhibition. Western blot assessments of p53 and actin in GSC#726/R1 cells treated with MG132 or DMSO (mock control). Increasing protein amounts (40, 60 and 80 µg per well) were loaded in lanes 1&4, 2&5 and 3&6, respectively.
